# Activated regulatory T cells suppress effector NK cell responses by an IL-2-mediated mechanism during an acute retroviral infection

**DOI:** 10.1186/s12977-015-0191-3

**Published:** 2015-07-30

**Authors:** Elisabeth Littwitz-Salomon, Ilseyar Akhmetzyanova, Cecilia Vallet, Sandra Francois, Ulf Dittmer, Kathrin Gibbert

**Affiliations:** Institute of Virology of the University Hospital in Essen, University of Duisburg-Essen, Essen, Germany

**Keywords:** NK cells, Friend retrovirus, Regulatory T cells, IL-2 consumption, Treg suppression

## Abstract

**Background:**

It is well established that effector T cell responses are crucial for the control of most virus infections, but they are often tightly controlled by regulatory T cells (Treg) to minimize immunopathology. NK cells also contribute to virus control but it is not known if their antiviral effect is influenced by virus-induced Tregs as well. We therefore analyzed whether antiretroviral NK cell functions are inhibited by Tregs during an acute Friend retrovirus infection of mice.

**Results:**

Selective depletion of Tregs by using the transgenic DEREG mouse model resulted in improved NK cell proliferation, maturation and effector cell differentiation. Suppression of NK cell functions depended on IL-2 consumption by Tregs, which could be overcome by specific NK cell stimulation with an IL-2/anti-IL-2 mAb complex.

**Conclusions:**

The current study demonstrates that virus-induced Tregs indeed inhibit antiviral NK cell responses and describes a targeted immunotherapy that can abrogate the suppression of NK cells by Tregs.

**Electronic supplementary material:**

The online version of this article (doi:10.1186/s12977-015-0191-3) contains supplementary material, which is available to authorized users.

## Background

Natural killer (NK) cells are innate effector lymphocytes that survey the body and destroy viral pathogens without previous sensitization. Activation of NK cells is mediated by a repertoire of competing activating and inhibitory signals of germline-encoded receptors. This dynamic balance between inhibitory and activating receptors allows NK cells to recognize and eliminate infected or tumor cells while leaving out normal unaltered cells. Activated NK cells produce proinflammatory cytokines such as interferon-γ (IFN-γ) or tumor necrosis factor-α. They can also induce apoptosis in target cells by the expression of cytotoxic molecules like granzymes (Gzm) or perforin or death receptor ligands on their cell surface including tumor necrosis factor related apoptosis inducing ligand (TRAIL) and Fas ligand. Potent activation and proliferation of NK cells depends on Interleukin (IL)-2, originally described as a T cell growth factor [1, 2]. IL-2 influences different lymphocyte subsets during immune responses, differentiation, survival and homeostasis. The IL-2 receptor is comprised of three subunits—alpha (CD25) [3], beta (CD122) and gamma (CD132) [4–6]. CD122 is broadly expressed on NK cells whereas CD25 is highly expressed on activated T cells, especially on a distinct CD4^+^ T cell population called regulatory T cells (Tregs) [4–6]. Tregs are crucial for maintaining peripheral tolerance, preventing autoimmune diseases and limiting chronic inflammation [7, 8]. However, by inhibition of virus-specific effector T cells the suppressive activity of Tregs can also be associated with progressive T cell exhaustion and establishment of chronic viral infections, as shown for human immunodeficiency virus (HIV) or hepatitis C virus infection of humans or the murine Friend retroviral (FV) infection [9–12]. Interestingly, Tregs do not merely suppress T cells but can also inhibit NK cell functions in autoimmune diseases, cancer and pregnancy [13, 14]. Particularly with regard to viral infections, NK cells contribute to the control of HIV-1 through recognition of infected cells by activating and inhibitory killer immunoglobulin-like receptors (KIRs) [15–18]. The important role of NK cells in controlling retroviral infections was demonstrated in studies showing that polymorphisms in KIRs as well as active suppression of ligand expression for the activating natural killer group 2 member D (NKG2D) receptor by HIV-1 resulted in reduced NK cell activity in HIV-1 infection [19, 20]. In our previous work, we also demonstrated an antiretroviral function of murine NK cells during the early phase of infection with FV [21].

It was recently described that Tregs control a subpopulation of CD127^+^ NK cells in naive mice [22]. Another study also showed Treg-mediated NK cell suppression during homeostasis, which was not seen during murine cytomegalovirus (CMV) infection [23]. However, to define the influence of Tregs on NK cell responses in viral infections, Tregs have to be experimentally depleted during an ongoing infection that induces potent Treg responses. We used the well-established FV mouse model, in which a strong activation and expansion of non-virus-specific natural Tregs occurs during the acute FV infection [24]. The FV complex consists of a replication-competent but apathogenic Friend murine leukemia virus and a pathogenic but replication-defective spleen focus-forming virus. Resistant mice, such as C57BL/6, are able to recover from acute infection, but develop a persistent infection [25]. During the acute phase of FV infection, antiviral CD8^+^ T cells are required for the control of viral replication. However, activated Tregs subsequently start to suppress the effector functions of CD8^+^ T cells, which contribute to the establishment of viral chronicity [9, 26–29].

Here, we investigated the suppressive capacity of virus-induced Tregs on NK cell responses during acute FV infection taking advantage of the transgenic DEREG mouse model in which Tregs can be selectively depleted by diphtheria toxin injection [30]. We identified a strong increase of NK cell maturation, activation and effector functions in Treg-depleted mice. Suppression by Tregs, which was mediated by IL-2 consumption, led to increased viral loads and reduced cytotoxicity of NK cells in vivo. This suppression could be overcome by application of IL-2/anti-IL-2 mAb complex, which specifically stimulated NK cells.

Thus, defined therapies with IL-2, which specifically improve NK cell responses, should be of interest to augment NK cell responses in viral infections or cancer.

## Results

### NK cell frequencies inversely correlate with viral loads in Treg-depleted mice

**Fig. 1:**
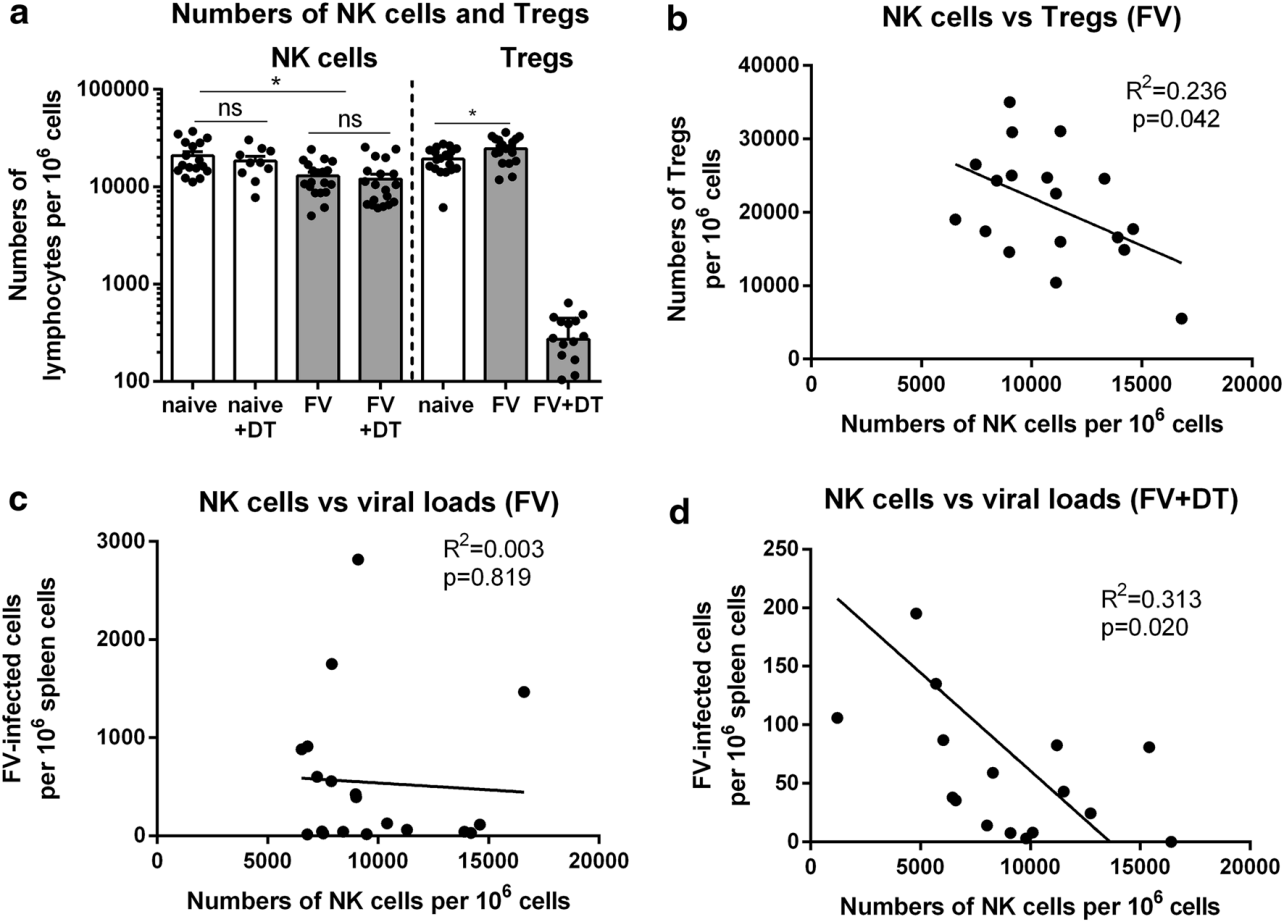
Correlation of NK cell frequencies with Treg frequencies and viral loads. DEREG mice were infected with FV and depleted for Tregs by repeated injections of DT. Uninfected DEREG mice were used as controls. Splenocytes were isolated at 12 dpi and proportions of NK cells (CD3^−^CD49b^+^NK1.1^+^) and Tregs (CD4^+^Foxp3^+^) were determined using flow cytometry (**a**,** b**). As a control numbers of Tregs following application of DT in transgenic DEREG mice were also shown (**a**, *right column*). Individual proportions and mean (±SEM) values are indicated by *bars* and *dots*. Viral loads of FV-infected mice (**c**) and FV-infected and Treg-ablated mice (**d**) were analyzed by infectious center assay and correlated to proportions of NK cells. A minimum of ten mice per group were used. Experiments were repeated at least three times. Statistically significant differences between the groups in **a** were determined by Kruskal–Wallis one-way analysis and Dunn’s multiple comparison tests (NK cells) or by using the unpaired student’s t test (Tregs) and are indicated by *single asterisk* for p < 0.05 and *ns* not significant. Statistically significant correlations were analyzed using the Pearson correlation test and results were shown in the graph.

In previous studies we showed that FV infection induced strong activation and expansion of Tregs during acute phase [12 days post infection (dpi)] of FV infection ([9]; Fig. 1a). These cells subsequently suppress CD8^+^ T cell responses [9]. Since no antiviral activity of NK cells could be demonstrated at the same time point [21], we determined if cytotoxic NK cells were suppressed by the expanded Tregs during acute FV infection. We infected C57BL/6 and DEREG mice with 20,000 spleen focus-forming units (SFFU) of FV and subsequently depleted up to 97% of Tregs by repeated injections of diphtheria toxin (DT). As control, Tregs were also depleted in non-infected mice. In contrast to other studies [22, 23], Treg depletion during homeostasis did not significantly change proportions of NK cells in the spleen (Fig. 1a), which might be due to the different time point we performed our analysis compared to recently published studies [22, 23]. At 12 dpi, when Treg frequencies peak in the spleen [9], we analyzed proportions of NK cells in this organ. Interestingly, FV infection resulted in reduced NK cells frequencies in the spleen and no expansion of NK cells after Treg depletion during acute FV infection was observed (Fig. 1a). However, we observed an increase in NK cell frequencies in lymph nodes and peritoneum (Additional file 1c, d). We also determined the frequencies of CD127^+^ NK cells, which were previously shown to be suppressed by Tregs in naive mice [22]. We did not detect any change in the frequency of this CD127^+^ NK cell subpopulation after Treg ablation (data not shown). Next, we examined whether the decreased proportions of NK cells in FV-infected mice correlated with Treg frequencies or viral loads. We observed an inverse correlation of Treg frequencies and NK cell frequencies in the spleen of FV-infected mice (Fig. 1b), whereas no correlation between NK cells and viral loads was detected at 12 dpi (Fig. 1c). This suggested that virus-induced Tregs might affect NK cell frequencies during acute FV infection. To prove this, we depleted Tregs by repeating injections of DT into FV-infected DEREG mice. In contrast to Fig. 1c we found an inverse correlation between frequencies of NK cells and viral loads after Treg depletion (Fig. 1d). These results suggest that Tregs might regulate NK cell responses during acute FV infection.

### NK cells show enhanced activation and effector molecule expression in the absence of Tregs

**Fig. 2:**
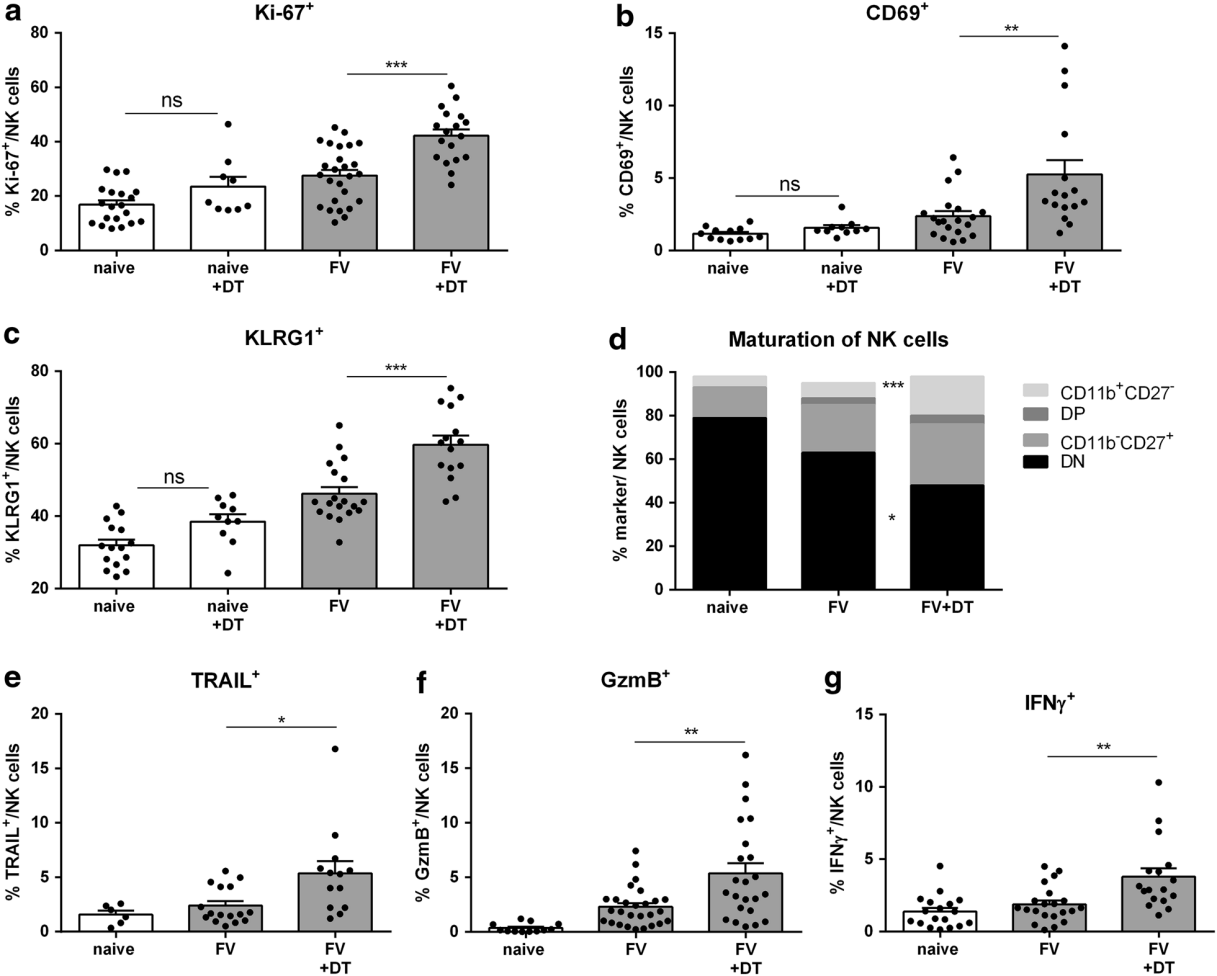
Proliferation, maturation and effector function of NK cells. DEREG mice were infected with FV and mice were Treg-depleted by repeated injections of DT. Uninfected DEREG mice were used as controls. At 12 dpi splenocytes were analyzed by flow cytometry. For the analysis of NK cells doublets were excluded and viable lymphocytes were determined. NK cells were identified from these cells as CD3^−^CD49b^+^NK1.1^+^ cells. The proliferation of NK cells was determined measuring the intracellular expression of Ki-67 (**a**) and the activation status was measured by surface expression of CD69 (**b**). The maturation profile was determined by surface expression of KLRG1 (**c**), CD11b and CD27 (**d**). The effector functions of NK cells were analyzed by surface expression of TRAIL (**e**) and intracellular expression of GzmB (**f**). Effector phenotype of NK cells was analyzed on basis of IFN-γ (**g**). Individual percentages and mean (±SEM) values are indicated by *dots* and *bars*. At least 13 mice per FV-infected group from at least two independent experiments were studied. Statistically significant differences between groups were analyzed by using the unpaired student’s t or Mann–Whitney test and are indicated by *single asterisk* for p < 0.05, *double asterisk* for p < 0.01, *triple asterisk* for p < 0.001 and *ns* not significant.

To further investigate the influence of Tregs on NK cell activation and function, we performed a phenotypic comparison of NK cells from FV-infected non-depleted and Treg-depleted mice (Fig. 2). Tregs can suppress NK cells during homeostasis [22, 23], therefore, we also used uninfected Treg-depleted and non-depleted mice as controls in the following experiments. As depicted in Fig. 2a, Additional file 2, as well as Additional file 1a and b, the proliferation of NK cells, measured by the intracellular expression of Ki-67 or the incorporation of BrdU into newly generated cells, was significantly increased in the absence of Tregs in FV-infected mice (42.17% Ki-67^+^ NK cells in Treg-depleted mice versus 27.5% Ki-67^+^ NK cells in non-depleted mice). We also determined if the proliferating NK cells underwent apoptosis by Annexin V staining, but we did not observe enhanced apoptosis of NK cells after Treg depletion (data not shown) indicating that newly generated NK cells (Ki67^+^ NK cells; BrdU^+^ NK cells) post Treg-depletion do not accumulate in the spleen, but rather in lymph nodes and the peritoneum (Additional file 1c, d). Analyzing proliferation, activation, or maturation of NK cells we did not find a significant change after depletion of Tregs in uninfected mice in comparison to non-depleted mice (Fig. 2a–d). In contrast, we detected a significant increase in the expression of the early activation marker CD69 (Fig. 2b; Additional file 2) and a significant downregulation of CD62L (data not shown), demonstrating their effector phenotype in Treg-deficient mice in comparison to non-depleted control mice during acute FV infection. Comparing the same experimental groups also significant higher percentages of KLRG1^+^ NK cells [31] (Killer cell lectin-like receptor subfamily G member 1), which represent mature NK cells, were observed in Treg-deficient mice (Fig. 2c; Additional file 2). To further characterize the differentiation state of NK cells, we analyzed the expression of the surface markers CD11b and CD27, which classify four main stages of NK cell development [32]. NK cells differentiate from immature CD11b^−^CD27^−^ (double negative, DN) to CD11b^−^CD27^+^ through CD11b^+^CD27^+^ (double positive, DP) to CD11b^+^CD27^−^ cells. The last two stages define mature NK cells (CD11b^+^CD27^+^ and CD11b^+^CD27^−^) and the latter is classified as most mature or terminally differentiated NK cells [32, 33]. After Treg ablation, we observed a significant decrease in the percentage of DN immature NK cells and a significant increase in CD11b^+^CD27^−^ terminally differentiated NK cells (18%) compared to non-depleted FV-infected mice (7%, Fig. 2d; Additional file 2). Similar results were obtained from an analysis of the effector phenotypes of NK cells. In the absence of Tregs, NK cells expressed significantly more TRAIL (Fig. 2e), GzmB (Fig. 2f) and IFN-γ (Fig. 2g) in comparison to FV-infected control mice. These results demonstrate a decreased NK cell proliferation, differentiation and effector function in FV-infected mice due to a virus-induced expansion of Tregs.

### Treg ablation results in potent target cell killing by NK cells

**Fig. 3:**
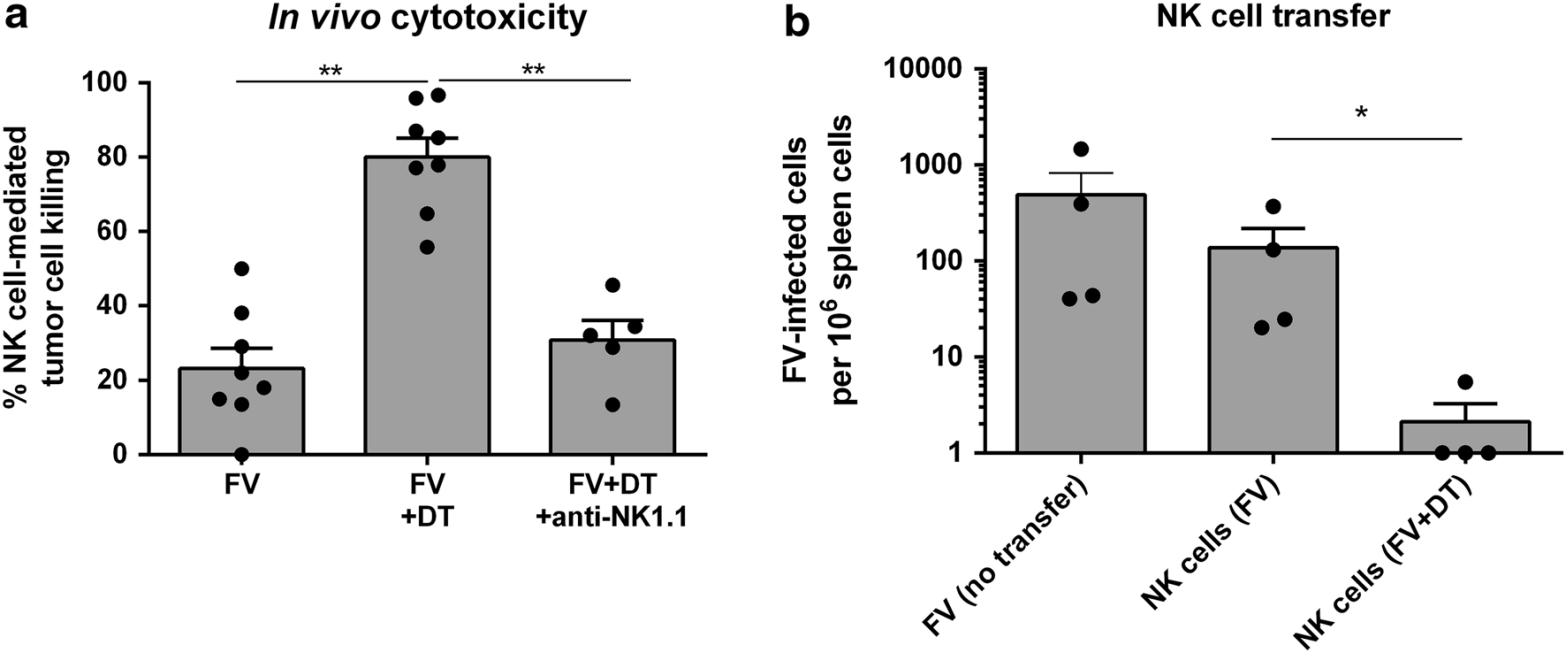
NK cell dependent target cell killing. DEREG mice were infected with FV and mice were either Treg-depleted by repeated injections of DT or NK cell-depleted or Treg- and NK cell-depleted. At 10 dpi mice received 5 × 10^5^ CFSE-labeled RMA/S. Two days later (12 dpi) cells were obtained by peritoneal lavage and analyzed by flow cytometry (**a**). At least three independent experiments were performed with at least five mice. Individual percentages and mean (±SEM) values were indicated by *dots* and *bars*.** b** DEREG mice were infected with FV and several mice were Treg-depleted by repeated injections of DT. At 10 dpi splenic NK cells were isolated and adoptively transferred into FV-infected mice. Four days post transfer mice were sacrificed and viral loads were analyzed in the spleen. FV-infected mice, which did not receive any cell transfer, were used as control. At least four mice per group were tested. Statistically significant differences between the different transferred NK cells were analyzed via Mann–Whitney test and are indicated by *single asterisk* for p < 0.05 and *double asterisk* for p < 0.01.

To investigate whether the increased expression of effector molecules on NK cells after Treg depletion in FV-infected mice correlates with a higher killing capacity of NK cells, we performed an in vivo NK cell cytotoxicity assay. Therefore, we transferred MHC class I-deficient tumor cells (RMA/S cells) into the peritoneal cavity of FV-infected mice at 10 dpi, where a similar increase in NK cell effector functions, representatively shown by TRAIL expression, was observed post Treg depletion (Additional file 1e). Two days later, we re-isolated the tumor cells by peritoneal lavage and counted the remaining CFSE-labeled RMA/S. In Treg-depleted mice up to 80% of the target cells were killed (Fig. 3a; FV+DT), whereas in Treg-containing mice only 23.2% of the RMA/S tumor cells had been eliminated by NK cells (Fig. 3a, FV). To prove that this killing was NK cell dependent, NK1.1^+^ cells were depleted in addition to Tregs (FV+DT+anti-NK1.1), which strongly reduced the killing to the same background level detected in control mice (FV). These results indicate that the general cytotoxicity of NK cells was strongly enhanced after Treg ablation in FV-infected mice. To analyze if this also translates into enhanced anti-FV activity of NK cells, we performed an adoptive transfer experiment with enriched NK cells (Fig. 3b). NK cells isolated from FV-infected Treg-depleted or non-depleted mice were transferred into FV-infected wild type mice. FV-infected mice without any cell transfer were used as controls. NK cells transferred from FV-infected donors did not significantly decrease viral loads compared to controls, whereas NK cells from Treg-deficient mice mediated an 80-fold reduction in viral loads (Fig. 3b). Here, we could show that Tregs suppress antiviral NK cell cytotoxicity during an acute retrovirus infection.

### Tregs deprive NK cells of IL-2 during acute FV infection

**Fig. 4:**
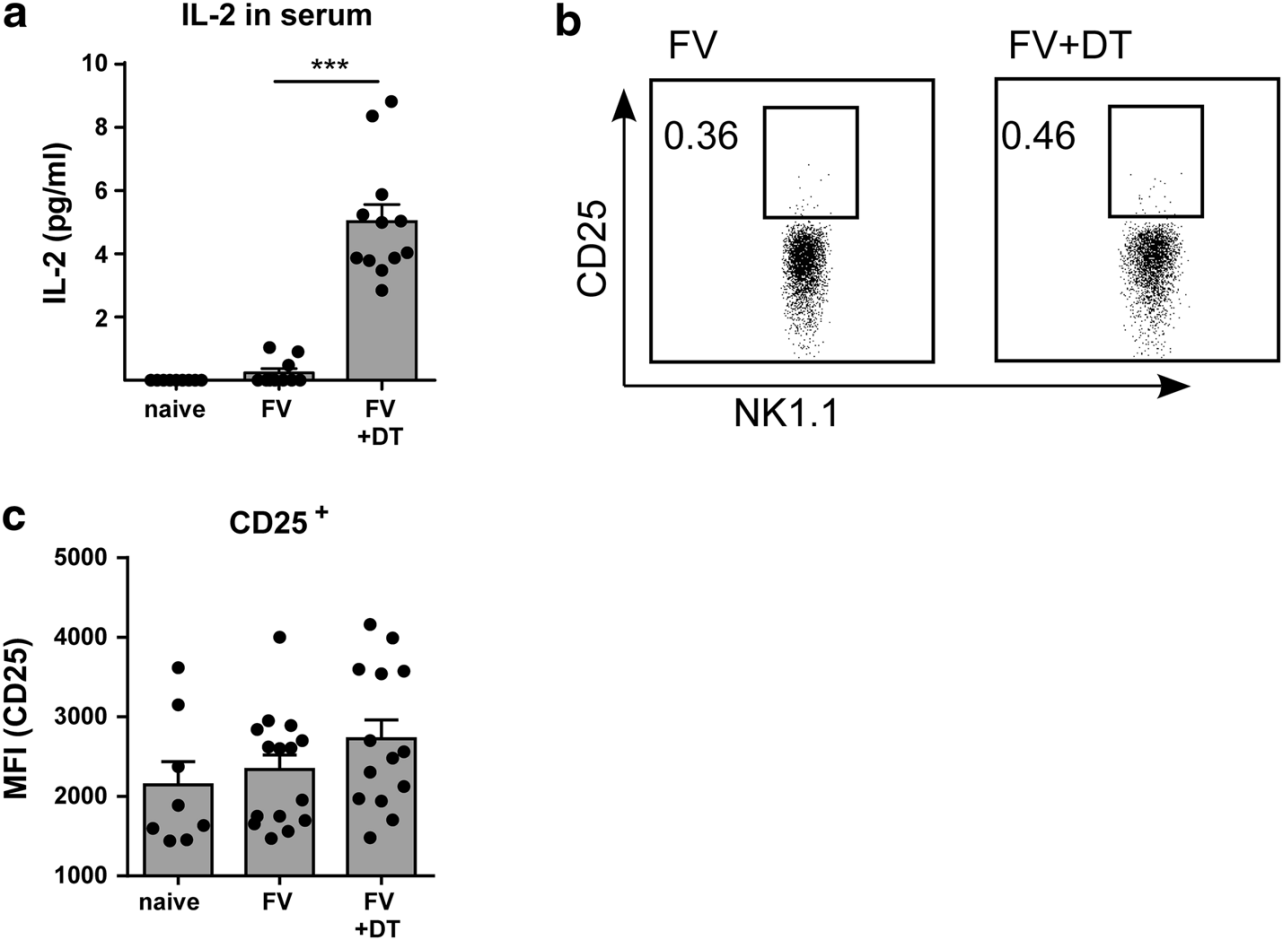
Effects of Treg ablation on IL-2 and IL-2R α-chain. DEREG mice were infected with FV and mice were Treg-depleted by repeated injections of DT. Uninfected DEREG mice were used as controls. Twelve days post infection blood and spleen were taken for analysis. Serum was analyzed for IL-2 concentration by ELISA (**a**). Samples of five different experiments were tested with at least ten samples per group. The increase of IL-2 concentration in serum is shown by *bars* and *dots* (±SEM). Statistically significant changes were determined by Mann–Whitney test and indicated by *triple asterisk* for p < 0.001. Representative *dot plots* of CD25 expression on splenic NK cells (NK1.1^+^ CD49b^+^ CD3^−^) in FV-infected and FV-infected and Treg-depleted mice are shown in (**b**). Expression of IL-2R α-chain (CD25) on NK cells was analyzed by flow cytometry and indicated by mean (±SEM) *values* and *dots* (**c**). A minimum of eight mice in at least two independent experiments were analyzed.

Next, we set out to analyze the mechanism of Treg-mediated suppression of NK cell responses during FV infection. Previous reports by Ghiringhelli et al. [34] and Smyth et al. [35] described a TGF-β-dependent suppression of NK cells by Tregs in cancer models. Hence, we analyzed TGF-β protein level in the serum and mRNA expression of TGF-β and also IL-10 in the spleen during FV infection, but we could not detect any differences in the expression levels between Treg-depleted and non-depleted mice (data not shown), suggesting that TGF-β and IL-10 do not play a role in our infection model. Then, we focused on IL-2, which influences different lymphocyte subsets, including Tregs and NK cells, in their proliferation, differentiation, and effector functions. To analyze the impact of IL-2 on NK cell responses during acute FV infection, we depleted Tregs by repeating injections of DT and measured the IL-2 concentration in the serum of mice. Uninfected Treg-depleted and DT-treated wild type mice served as controls, but we could not measure significant increased levels of IL-2 in the serum of these mice (data not shown). However, we detected a 21-fold increase in mean IL-2 serum levels between FV-infected mice with Tregs and FV-infected mice lacking Tregs (Fig. 4a), suggesting that Tregs consume large amounts of IL-2 or they suppress the production of IL-2 by CD4^+^ T cells [36].

IL-2 binds to the high-affinity trimeric IL-2 receptor (CD25, CD122 and CD132) and to the low affinity dimeric IL-2 receptor consisting of CD122 and CD132 [37]. Tregs express preferably the trimeric IL-2 receptor, whereas dimeric receptors are rather associated with CD8^+^ T cells and NK cells. Treg depletion did not change the proportion of CD122^+^ NK cells in comparison to non-depleted mice (data not shown) during FV infection. Analysis of the IL-2R α-chain (CD25) also revealed no changes in the expression levels after Treg depletion in uninfected and FV-infected mice (Fig. 4b, c).

### Specific IL-2 stimulation of NK cells mimics the effect of Treg depletion on NK cell activation and differentiation and leads to better FV control

**Fig. 5:**
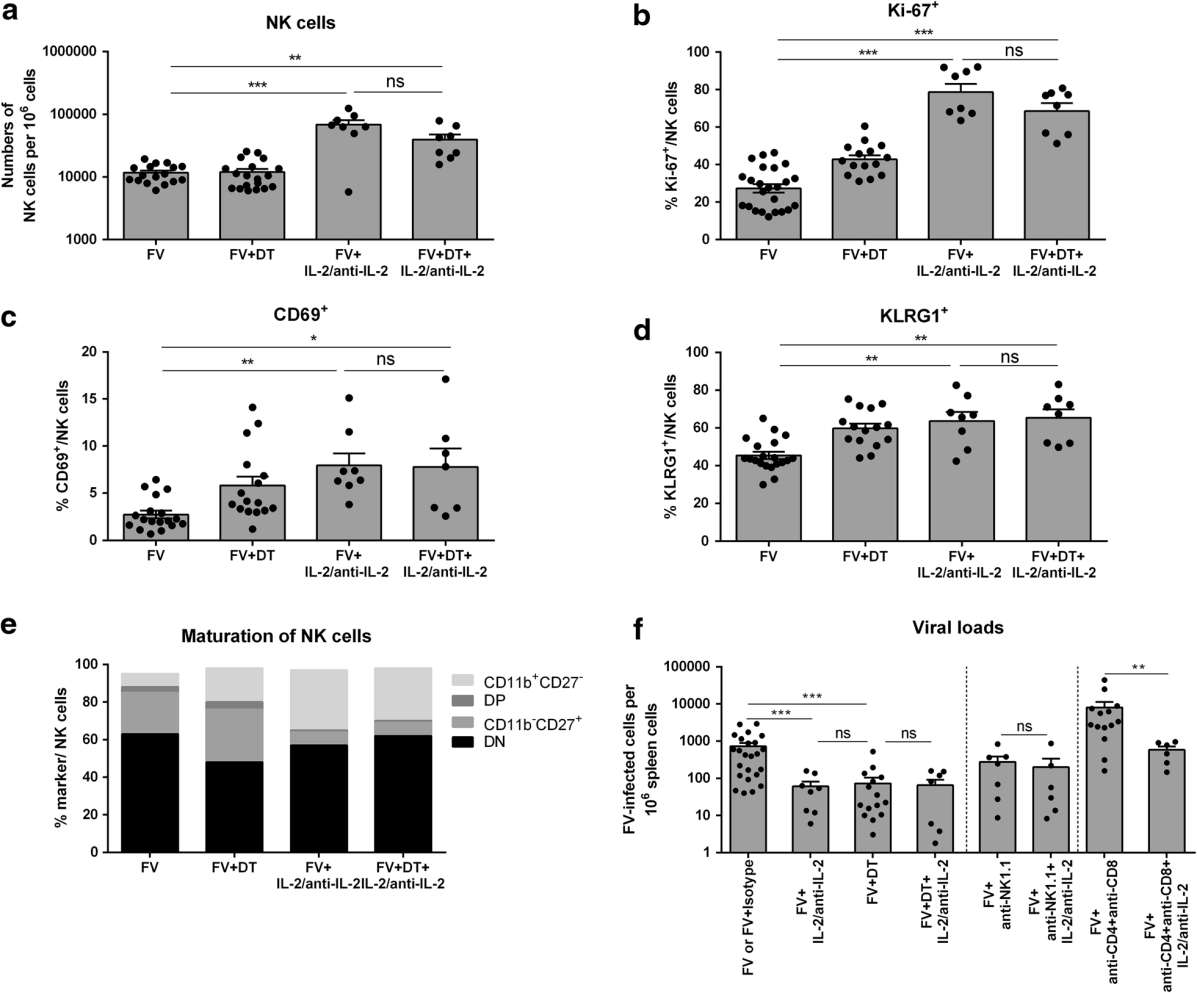
Influence of IL-2 treatment on NK cell effector function and FV control. Mice were infected with FV and several mice were either Treg-depleted by repeated injections of DT and/or stimulated with IL-2/anti-IL-2 mAb complex to specifically stimulate NK cells. At 12 dpi frequencies of splenic NK cells were determined by flow cytometry (**a**). The proliferation of NK cells was measured by the intracellular expression of Ki-67 (**b**) and the activation of NK cells was analyzed by surface expression of CD69 (**c**). The maturation profile was determined by surface expression of KLRG1 (**d**), CD11b and CD27 (**e**). Statistically significant differences between the three groups in **a**–**d** were determined either by Kruskal–Wallis one-way analysis and Dunn’s multiple comparison tests or by the ordinary one-way ANOVA. Statistically significant differences in** e** analyzed by Mann–Whitney test could be detected between FV and both treated groups (CD27^−^CD11b^+^ **p < 0.01, CD27^+^CD11b^−^ **p < 0.01), but no significant differences were detectable between IL-2/anti-IL-2 mAb complex-treated groups. At least seven mice per group from at least two independent experiments were used and indicated by mean (±SEM) *values* and *individual dots*.** f** Viral loads during FV infection and treatment with either IL-2/anti-IL-2 mAb complex or isotype control were analyzed by infectious center assay. NK cells were depleted by injections of supernatant fluid containing NK1.1-specific monoclonal antibody PK136. Viral loads were analyzed in NK cell-depleted and IL-2/anti-IL-2 mAb complex treated mice. FV-infected mice were also depleted of CD4^+^ and CD8^+^ T cells or Tregs and several mice were additionally stimulated with IL-2/anti-IL-2 mAb complex. Results are indicated by mean (±SEM) *values* and *dots*. At least six mice per group were analyzed in two independent experiments. Statistically significant differences within the T cell-competent groups (FV/FV+Isotype, FV+IL-2/anti-IL-2) and the T cell-depleted groups (FV+DT, FV+DT+IL-2/anti-IL-2, FV+anti-CD4+anti-CD8, FV+anti-CD4+anti-CD8+IL-2/anti-IL-2) were tested using Mann–Whitney test and indicated by *single asterisk* for p < 0.05, *double asterisk* for p < 0.01 and *triple asterisk* for p < 0.001 and *ns* not significant.

To confirm that IL-2 is an important factor in Treg-mediated suppression of NK cell responses, we specifically stimulated NK cells with IL-2 using a complex of anti-mouse IL-2 mAb (clone S4B6-1) in combination with recombinant mouse IL-2 protein. This antibody binds to the site of IL-2, which normally interacts with CD25 and thus boosts binding of IL-2 to the two other receptor subunits (CD122 and CD132) [37, 38], which are highly expressed on NK cells but not on Tregs. Application of IL-2 complex resulted in an increase in NK cell frequencies (FV+IL-2/anti-IL-2) compared to control mice (FV) (Fig. 5a), which was also seen in the expression of the proliferation marker Ki-67 (Fig. 5b). However, an additional depletion of Tregs (FV+DT+IL-2/anti-IL-2) did not further change the total frequencies of NK cells in comparison to IL-2 stimulated FV-infected mice (FV+IL-2/anti-IL-2).

We also detected a significant increase in the expression of the early activation marker CD69 (Fig. 5c) and the maturation marker KLRG1 (Fig. 5d) after stimulation with the IL-2/anti-IL-2 mAb complex which were comparable to those seen in Treg-depleted mice. The combination of both treatments (FV+DT+IL-2/anti-IL-2) did not further increase the expression of both markers (Fig. 5c, d).

Next, we analyzed the differentiation state of NK cells after stimulation with IL-2 by expression of the surface molecules CD11b and CD27 (Fig. 5e). We observed a massive increase in CD11b^+^CD27^−^ terminally differentiated NK cells (32%) compared to control mice (7%), which was also seen after Treg depletion (FV+DT). This effect is not further increased by additional Treg depletion (FV+DT+IL-2/anti-IL-2), suggesting that NK cells are saturated with IL-2 after application of the IL-2/anti-IL-2 mAb complex. Additional depletion of Tregs might increase the level of circulating IL-2, which is not further consumed by stimulated NK cells.

Finally, we wanted to determine whether the improved NK cell activation by IL-2 treatment has an effect on viral loads. Interestingly, specific NK cell stimulation with IL-2 in the presence of Tregs mediated a significant reduction in viral loads (FV+IL-2/anti-IL-2; Fig. 5f), which was comparable to the results seen in Treg-depleted mice (FV+DT; Fig. 5f). In line with our previous results of NK cell activation and effector function (Fig. 5b–e), the combination of Treg depletion and NK cell stimulation did not further decrease viral burden (FV+DT+IL-2/anti-IL-2; Fig. 5f). To confirm that the observed reduction in the viral loads post IL-2 treatment is mediated by NK cells and not by other cells which express the IL-2 receptor, we depleted CD8^+^ and CD4^+^ T cells by injection of supernatant fluid containing CD8-specific mAb 169.4 and CD4-specific mAb YTS 191.1. We stimulated NK cells by application of IL-2/anti-IL-2 mAb complex in these T cell depleted mice and determined viral loads at 12 dpi. As expected, after depletion of CD4^+^ and CD8^+^ T cells, the viral loads significantly increased (FV+anti-CD4+anti-CD8; Fig. 5f; Additional file 3), as T cells, especially CD8^+^ T cells, are required for FV control at that time point [9]. An additional NK cell depletion resulted in a further rise of the viral loads (Additional file 3) demonstrating that NK cells are involved in antiviral immunity during acute FV infection. In contrast, specific NK cell stimulation with IL-2 in T cell-depleted mice (FV+anti-CD4+anti-CD8+IL-2/anti-IL-2) led to a more than 13-fold reduction in the viral loads compared to unstimulated T cell-depleted controls (FV+anti-CD4+anti-CD8) showing that IL-2-stimulated NK cells contribute to FV control.

Additionally we injected IL-2/anti-IL-2 mAb complexes (clone JES-1) targeting Tregs to clarify that specific IL-2 stimulation of NK cells is required for FV control. In contrast to the antibody complexes used before, application of the other IL-2 antibody complex specifically activated Tregs (expression of CD43; Additional file 4c) and this did not result in reduction of viral loads (data not shown).

We also analyzed the activation status of Tregs, CD4^+^ and CD8^+^ T cells after treatment with IL-2/anti-IL-2 mAb complexes, but we did not detect any effect of IL-2 on T cells compared to FV-infected control mice (Additional file 4a, b). Furthermore, NK cell depletion during IL-2/anti-IL-2 mAb complex application completely abolished IL-2-mediated antiviral immunity (Fig. 5f). This implies that the therapeutic effect of IL-2 was mediated by NK cells and could overcome the suppressive effect of virus-induced Tregs, which led to control of acute FV infection. Here, we describe a new suppressive mechanism of Tregs on NK cell functions during an acute viral infection that is dependent on IL-2 availability or IL-2 consumption by Tregs.

## Discussion

Efficient immune protection against pathogens such as viruses is usually provided by a successful interaction of innate and adaptive immunity. NK cells participate in the rapid control of early viral replication, whereas virus-specific T cells undergo clonal expansion and thus contribute later to antiviral immunity. A potent immune response has to be counter-regulated to prevent immunopathology. This immune control is efficiently mediated by Tregs, which dampen pathogen-specific effector T cell responses. Besides preventing immunopathology, Treg activity can also contribute to inefficient clearance of pathogens and the establishment of chronic viral infections [39]. However, whether Tregs also influence the initial antiviral response of NK cells was not known.

The current study shows for the first time that NK cell responses are suppressed by virus-induced Tregs during an acute retroviral infection. Specific depletion of Tregs during acute FV infection resulted in improved NK cell maturation, differentiation and effector functions (Fig. 2). The suppression of NK cells was mediated by Treg-driven IL-2 consumption, which can be overcome by specific stimulation of NK cells with an IL-2/anti-IL-2 mAb complex in vivo. Others showed a Treg-mediated suppression of NK cell responses in cancer, autoimmunity or during homeostasis, whereas no studies on Treg-mediated NK cell suppression in viral infections existed. Only one recent study using CMV-infected mice analyzed the impact of Treg depletion on NK cell responses. Lindenberg and colleagues [23] showed that during acute mouse CMV infection Tregs suppress only T cell responses, whereas NK cell responses were not affected by Tregs. However, CMV infection of mice is not a very useful model to study the interaction of Tregs and NK cells because the virus does not efficiently activate or expand Tregs [40]. The effect of virus-induced Tregs on anti-viral NK cell responses can only be investigated in an infection model with strong Treg responses, like the FV mouse model [9, 41]. In a mouse cancer model, CD4^+^CD25^+^ Tregs inhibited NKG2D-mediated NK cell cytotoxicity by TGF-β release and subsequent tumor rejection and metastasis inhibition [35]. Similar results were also shown by Ghiringhelli and colleagues [34] who described suppression of NK cell cytotoxicity by Tregs in a mouse B16 tumor model and human melanoma patients. Our current data and the described studies imply that negative regulation of NK cell responses by Tregs might be a common feature in infections and cancer. Besides cytotoxic T cells, also NK cell cytotoxicity is controlled by Tregs most likely to avoid immunopathology.

Several molecular mechanisms of Treg suppression of NK cells have been discussed. One mechanism used by Tregs to suppress antitumor responses depends on granzyme B and perforin and thus on direct killing of NK cells and CD8^+^ T cells by Tregs in allogenic B16 and RMA/S tumors [42]. However, in FV-infected mice, no expression of granzymes has been found in activated Tregs [43], suggesting that other mechanisms of suppression play a more important role. As TGF-β is an important factor in Treg-mediated suppression of NK cells in cancer models [34, 35], we also analyzed the expression of TGF-β in FV-infected and additionally Treg-depleted animals. We did not detect any differences after depletion of Tregs (data not shown), indicating that TGF-β is not involved in the suppression of antiviral NK cell effector functions during FV infection.

In the mouse B16 melanoma model described above, the authors also demonstrated that an in vitro stimulation of PBMC cultures with IL-2 neutralized the Treg-mediated suppression of NK cells suggesting an important role of IL-2 in Treg-NK cell interaction [34]. Our findings and recent studies suggest a cellular interplay of Tregs, CD4^+^ helper T cells and NK cells. Tregs do not produce IL-2 by themselves, but they consume IL-2 which is mainly produced by CD4^+^ T cells. The hypothesis is that Tregs dampen the bioavailability of IL-2 by massive consumption of the cytokine or inhibition of IL-2 production by CD4^+^ T cells and thus indirectly inhibit NK cells due to IL-2 deprivation. There is evidence from a mouse diabetes model showing the importance of Treg suppression and IL-2 consumption in an autoimmune disease [44]. Here, Tregs limited the availability of IL-2 in the pancreatic islets, which dampened activation and effector functions of infiltrating NK cells. However, in the absence of Tregs, IL-2 produced by infiltrating CD4^+^ helper T cells activated NK cells and enhanced their IFN-γ production which leads to the onset of diabetes. This effect was prevented by application of an IL-2 antagonist in Treg-depleted mice demonstrating the importance of IL-2 in regulating NK cell functions. Others found that depletion of Tregs in Foxp3.DTR mice did not change NK cell tolerance to self- or non-self-ligands, whereas missing-self-induced cytotoxicity of NK cells was enhanced in Treg-depleted mice. They showed that this was dependent on CD4^+^ helper T cells producing IL-2 and that regulating IL-2 levels may be a mechanism of Tregs to tune NK cell activity [45]. During acute FV infection, IL-2 is mainly produced by CD4^+^ helper T cells [24, 36], which was subsequently consumed by expanded Tregs. NK cells only received sufficient IL-2 signaling for full activation when the IL-2 consumption by Treg was limited. Thus, our previous data and the current results suggest an interplay of CD4^+^ helper T cells, Tregs and NK cells also in viral infections with IL-2 being the specific link between these cell populations. Gasteiger et al. [22] described that Tregs mainly control a very specific NK cell subpopulation (CD127^+^) during homeostasis, which was also dependent on the availability of IL-2. However, this population of NK cells does not seem to play a major role during FV infection, as it accounted for only 0.5% of all NK cells in infected mice, and this frequency did not change after depletion of virus-induced Tregs.

Immunotherapy with IL-2 is already used in the clinics to treat patients with metastatic melanoma and renal cell carcinoma with objective clinical response rates of 15–25%. As IL-2 has multiple activities on various lymphocytes, treatment with IL-2 results in a variety of different side effects. Many trials have been performed to reduce these by changing the dose, route, or chemical modification of IL-2 or adding additional immunomodulators; however, they were not successful in enhancing the clinical responses [46]. Another attempt is to selectively target distinct cell populations by IL-2 immunotherapy. The IL-2 receptor consists of three subunits: CD25 (α), CD122 (β) and CD132 (γ) [38]. CD25 is highly expressed on Tregs, but hardly expressed on NK cells and memory-phenotype CD8^+^ T cells [47]. Addition of anti-IL-2 mAb to recombinant IL-2 can target the cytokine to specific cell subsets. In mice, two different clones of anti-IL-2 mAb are available that bind to different sites of the cytokine. Clone JES6-1 shields the CD122 binding site which further targets this antibody to CD25, whereas clone S4B6 interferes with the CD25 binding site of IL-2 and thus leads to preferential CD122 binding [38, 47]. Application of one of these antibodies together with recombinant IL-2 can therefore target IL-2 to Tregs (clone JES6-1) or to NK cells or memory-phenotype CD8^+^ T cells (clone S4B6). In our study, we used clone S4B6 to specifically stimulate NK cells and reduce binding to Tregs. We observed a significant activation, maturation and differentiation of NK cells upon this IL-2 immunotherapy (Fig. 5), whereas no stimulatory effect was detected on different T cell subsets (Additional file 2). The selective IL-2 stimulation of NK cells reduced viral loads (Fig. 5f) and could overcome suppressive Treg activity suggesting that this might be an interesting new approach for the treatment of infectious diseases. Studies in a B16 melanoma model have shown that immunotherapy with an IL-2/anti-IL-2 mAb complex only binding to the IL-2βγ receptor abrogated IL-2 therapy mediated side effects (pulmonary edema) and led to a vigorous activation of cytotoxic immune cells with strong antitumor reactivity [5]. In their mouse model for autologous hematopoietic stem cell transplantation, Newman and colleagues demonstrated that vaccination with lymphoma cells secreting antibodies in combination with IL-2/anti-IL-2 mAb complexes (clone S4B6) induced strong antitumor responses of cytotoxic cells (CD8^+^ T cells and NK cells) and thus prevented the establishment of hematologic cancers [48]. Others compared the efficacy of the IL-2/anti-IL-2 mAb complex with IL-2 treatment alone in a B16 melanoma mouse model. The combined treatment could markedly enhance cytotoxicity of CD8^+^ T cells and partially protect mice from tumor metastasis compared to IL-2 treatment alone [49]. Application of IL-2/anti-IL-2 mAb (clone S4B6) complexes strongly extends the half-life of soluble IL-2 and prevents binding to CD25 on Tregs resulting in a potent improvement of the biological activity of the complex compared to soluble IL-2 [47]. However, these studies did not discuss the role of Tregs in their infection models [50].

Similar antibodies for IL-2 to target different cell subsets also exist for the use in humans. Complexes of human IL-2 and anti-IL-2-mAB clone MAB602 closely resemble S4B6-IL-2 complexes targeting IL-2 to CD122 on the surface of immune cells. An equivalent for JES6-1 directing IL-2 to CD25 (mAb 5344) does also exist. Also, two mutated isoforms of human IL-2 were developed with increased binding to either CD25 or CD122 [51, 52] implying that targeted IL-2 immunotherapies in humans may be feasible in the future.

To our knowledge, this is the first study showing that immunotherapy with an IL-2/anti-IL-2 mAb complex can overcome Treg suppression and improve NK cell responses in an acute retroviral infection. The specific stimulation of NK cells by IL-2 can result in improved control of viral infections. Such a therapeutic approach has a clear advantage over Treg depletion or manipulation during viral infections, as it may significantly reduce the risk of inducing immunopathologies. Using antibody–cytokine-complexes to specifically target distinct immune cell populations may have a strong impact in future therapies against infections and cancer, as side-effects in patients can be reduced and clinical responses improved.

## Conclusion

Natural killer (NK) cells, which belong to the innate arm of the immune system, can contribute to the control of many viral infections. NK cells can directly kill virus-infected cells and thus reduce viral replication. However, in infections with retroviruses, like HIV, NK cells are not sufficient to prevent pathology. Here, we showed that NK cell effector functions are strongly inhibited by virus-induced regulatory T cells leading to failure of NK cells to control retroviral infection. We described IL-2-mediated suppression mechanism by regulatory T cells which can be overcome by NK cell-specific stimulation with IL-2 complexes. This new therapeutic approach to target specific immune cells by antibody–cytokine-complexes might have great potential for the treatment of many viral infections or cancer.

## Methods

### Ethics statement

Animal experiments were performed in strict accordance with the German regulations of the Society for Laboratory Animal Science (GV-SOLAS) and the European Health Law of the Federation of Laboratory Animal Science Associations (FELASA). The protocol was approved by the North Rhine-Westphalia State Agency for Nature, Environment and Consumer Protection (LANUF) (Permit Number: G1341/12). All efforts were made to minimize suffering.

### Mice and virus

Experiments were done using sex- and age-matched inbred C57BL/6 (B6, Harlan Laboratories, Germany) and wild type and transgenic DEREG mice [30]. At the beginning of the experiments, mice were 6–10 weeks old. The FV stock used in these experiments was a FV complex containing B-tropic Friend murine leukemia helper virus and polycythemia-inducing spleen focus-forming virus. The stock was prepared as a 15% spleen cell homogenate from BALB/c mice infected 14 days previously with 3,000 SFFU of FV. Mice were injected intravenously with 0.15 ml phosphate-buffered saline (PBS) containing 20,000 SFFU of FV. The virus stock did not contain lactate dehydrogenase-elevating virus. Mice were sacrificed at 12 dpi by cervical dislocation.

### Detection of FV-infected cells in spleen

Infectious centers (IC) were detected by tenfold dilutions of single-cell suspensions onto *Mus dunnis* cells. Cultures were incubated for 3 days, fixed with ethanol, stained with F-MuLV envelope-specific monoclonal antibody 720 and developed with peroxidase-conjugated goat anti-mouse antibody and aminoethylcarbazol to detect foci [53].

### IL-2 concentration in the serum

For detection of IL-2 concentration in FV-infected mice, sera were harvested at 12 dpi. Cytokine concentration was measured by an IL-2 ELISA (eBioscience) according to the manufacturer’s protocol.

### Flow cytometry staining

Cell surface staining was performed using the following antibodies: anti-CD3 (17A2, eBioscience), anti-CD4 (GK1.5, eBioscience), anti-CD8 (53-6.7, eBioscience), anti-CD11b (M1/70, BD Bioscience), anti-CD25 (PC61, BD Bioscience), anti-CD27 (LG.3A10, BioLegend), anti-CD43 (1B11, BioLegend), anti-CD49b (DX5, eBioscience), anti-CD69 (H1.2F3, eBioscience), anti-CD127 (AFR34, eBioscience), anti-KLRG1 (2F1, eBioscience), anti-NK1.1 (PK136, BD Bioscience), anti-TRAIL (N2B2, eBioscience). Dead cells were excluded from analysis via fixable viability dye (eBioscience). For intracellular IFN-γ staining, cells were stimulated with Ionomycin (500 ng/ml), PMA (25 ng/ml) and Brefeldin A (2 μg/ml) and incubated for 3 h at 37°C. For intracellular staining of IFN-γ (XMG1.2, eBioscience) and GzmB (clone GB11, Life technologies), cells were fixed and permeabilized with CytoFix/CytoPerm (BD Bioscience) for 10 min. Intranuclear fixation was performed following the manufacturer’s instruction (Foxp3/Transcription Factor Fixation/Permeabilization, eBioscience). Tregs were stained with antibodies against Foxp3 (FJK-16S, eBioscience) and proliferation was detected using Ki-67 (SolA15, eBioscience). Data were acquired on LSR II flow cytometer (BD Bioscience) and analyses were performed using FACSDiva (BD Bioscience) and Flow Jo (Tree Star, USA) software.

### BrdU treatment and staining

5-Bromo-2′-deoxyuridine (BrdU, Sigma) was added to the drinking water of mice and was replaced every day (40 mg). In addition, mice were injected with BrdU every day starting at day 9 (i.p., 1 mg). BrdU staining was performed with BrdU Flow Kit (BD Pharmingen) according to the manufacturer’s protocol. Staining of cells was done with anti-BrdU (Clone BU20A, eBioscience) and acquired on LSR II flow cytometer (BD Bioscience).

### IL-2 receptor stimulation

Specific stimulation of NK cells were obtained by intraperitoneal inoculation of 50 µg anti-mouse IL-2 monoclonal antibody (Clone S4B6-1 or JES6-1, BioXCell) and 1 µg of recombinant mouse IL-2 (carrier-free, eBioscience) in 500 µl of PBS. Control groups received 50 µg of isotype control (rat IgG2a, BioXCell). Mice were injected every other day starting from day 7 to day 11 post infection.

### NK cell transfer

Splenic NK cells from FV-infected mice were enriched using NK cell isolation Kit II (Miltenyi Biotec) according to the manufacturer’s protocol. Intravenous injection of 2 × 10^6^ NK cells was performed at 10 dpi and viral loads were analyzed 4 days later. Transferred cells contained more than 85% of NK cells and only 2% of T cells.

### Depletion of T cells

For depletion of CD8^+^ T cells mice were injected intraperitoneally with 0.35 ml supernatant fluid containing CD8-specific mAb 169.4 every other day. Intraperitoneal injections of 0.15 ml CD4-specific mAb YTS 191.1 specifically deplete the CD4^+^ T cell population. At the days of analysis more than 98% of CD4^+^ T cells and at least 90% of CD8^+^ T cells were depleted in the spleen. To deplete regulatory T cells transgenic DEREG mice were injected intraperitoneally with DT (0.5 μg, Merck Millipore) diluted in PBS every third day for three times starting at day 5 post infection. The treatment depleted over 97% of CD4^+^ eGFP^+^ T cells in the spleen. Wild type DEREG mice injected with DT were used as controls.

### NK cell depletion

NK cell depletion was performed as described before [21]. At the day of analysis more than 95% of NK cells (CD3^−^ CD49b^+^ NK1.1^+^) were depleted in the spleen.

### In vivo NK cell cytotoxicity assay

In vivo NK cell cytotoxicity assay was performed using 5 × 10^5^ RMA/S cells per mouse labeled with 10 µM of CFSE (Vybrant^®^ CFDA SE Cell Tracer Kit, Life technologies). RMA/S cells were injected intraperitoneally at 10 dpi. NK cell-depleted mice were used as controls. Mice were sacrificed at 12 dpi and intraperitoneal lavage was performed with 10 ml PBS to obtain cells. Cells were washed once, resuspended in buffer containing fixable viability dye (eBioscience) to exclude dead cells and analyzed by flow cytometry. Target cell killing was calculated as follows:

$$\frac{{\text{Target cells from NK cell depleted mice } - \text{Sample target cell number}}}{\text{Target cells from NK cell depleted mice}} \times 100$$

### Statistical analyses

Statistical analyses and graphical presentations were computed with Graph Pad Prism version 6. Normal distribution was assessed using D’Agostino–Pearson omnibus test. Statistical differences between two different groups were determined by the unpaired student’s t test (parametric distribution) or Mann–Whitney test (non-parametric distribution). Analyses including several groups were tested using Kruskal–Wallis one-way analysis of variance on ranks and Dunn’s multiple comparison test (non-parametric distribution) or the ordinary one-way ANOVA and Bonferroni’s multiple comparisons test (parametric distribution). For the determination of correlating variables, linear regression and Pearson correlation were performed.

## Additional files

Additional file 1: Numbers, proliferation and effector functions of NK cells during FV infection DEREG mice were infected with 20.000 SFFU of FV for 12 days. (a, b) Mice were treated with 5-Bromo-2′-deoxyuridine (BrdU) starting at day 5 post FV infection. Splenocytes were isolated and NK cells (NK1.1+CD49b+CD3− cells) stained for flow cytometry. NK cells were analyzed for BrdU+ expression (a, b) and a representative graph of BrdU+ NK cell counts is shown in (a). Numbers of NK cells were analyzed in lymph nodes (c) and peritoneum (d). Representative dot plots were shown for TRAIL expression in the peritoneal lavage of FV-infected and FV-infected and Treg-depleted (DT) mice (e). Results are indicated by bars and dots +SEM. At least four mice per group were analyzed. Statistically significant differences between groups were analyzed by Mann–Whitney test and indicated by * for p < 0.05 and ** for p < 0.01. Additional file 2: Representative dotplots of activated, matured and proliferated NK cells Splenocytes were isolated from FV-infected mice (12 dpi) and NK cells were analyzed by flow cytometry (NK1.1+CD49b+CD3− cells). NK cells were evaluated for the different, indicated markers (Ki-67, CD69, KLRG1, CD27, CD11b). In the first row, representative dot plots of NK cell markers are shown during FV-infected. In the middle, dot plots of NK cells from FV-infected as well as Treg-ablated mice are depicted. Fluorescence-minus-one controls (FMOs) were performed for every staining (third row). Additional file 3: Viral loads in T cell- and NK cell-depleted mice during FV infection DEREG mice were infected with 20.000 SFFU of FV and either depleted for CD4+ and CD8+ T cells or NK cell-depleted. At 12 dpi viral loads were analyzed in spleens via infectious center assay. Individual proportions and mean (±SEM) values are indicated by dots and bars. At least 14 mice per group were analyzed in three independent experiments. Statistically significant differences between groups were analyzed by Kruskal–Wallis test and indicated by *** for p < 0.001. Additional file 4: Influence of IL-2/anti-IL-2 mAb complex treatment on T cell subsets Mice were infected with 20.000 SFFU of FV and sacrificed at 12 dpi. If indicated, Tregs were depleted by repeated injections of DT and mice were stimulated by injections of IL-2/anti-IL-2 mAb complex. Control mice were inoculated with isotype control. Single cell suspensions of splenocytes were stained for characteristic T cell markers ((a) CD4+ T cells; (b) CD8+ T cells and (c) Tregs) as well as activation (CD43) was analyzed using flow cytometry. Tregs were also stimulated with IL-2/anti-IL-2 mAb complex targeting CD25 on cell surface of Tregs (JES6-1) (c). Dotted lines represent the mean activation in naive mice. At least four mice per group were analyzed. Statistically significant differences between FV and FV+IL2/anti-IL-2 (JES6-1) were analyzed by Mann-Whitney test and indicated by * for p < 0.05.
