# Supplementary figures and images for: Activated regulatory T cells suppress effector NK cell responses by an IL-2-mediated mechanism during an acute retroviral infection
Source: Retrovirology. 2015 Jul 30;12:66. doi: 10.1186/s12977-015-0191-3 (PMC4518534; doi:10.1186/s12977-015-0191-3)

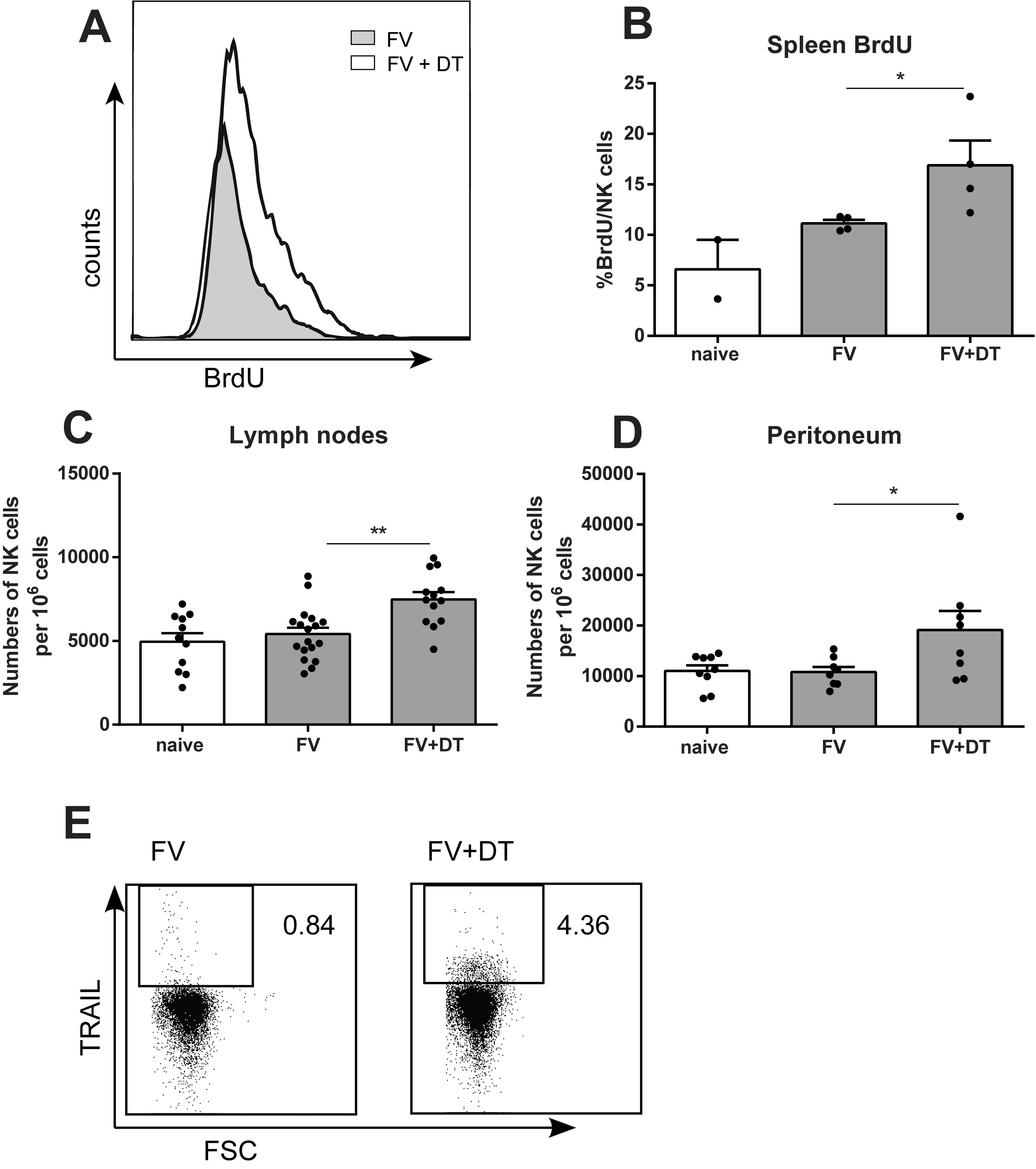

Supplement: Additional file 1: — Numbers, proliferation and effector functions of NK cells during FV infection DEREG mice were infected with 20.000 SFFU of FV for 12 days. (a, b) Mice were treated with 5-Bromo-2′-deoxyuridine (BrdU) starting at day 5 post FV infection. Splenocytes were isolated and NK cells (NK1.1+CD49b+CD3− cells) stained for flow cytometry. NK cells were analyzed for BrdU+ expression (a, b) and a representative graph of BrdU+ NK cell counts is shown in (a). Numbers of NK cells were analyzed in lymph nodes (c) and peritoneum (d). Representative dot plots were shown for TRAIL expression in the peritoneal lavage of FV-infected and FV-infected and Treg-depleted (DT) mice (e). Results are indicated by bars and dots +SEM. At least four mice per group were analyzed. Statistically significant differences between groups were analyzed by Mann–Whitney test and indicated by * for p < 0.05 and ** for p < 0.01. [file 12977_2015_191_MOESM1_ESM.tif]

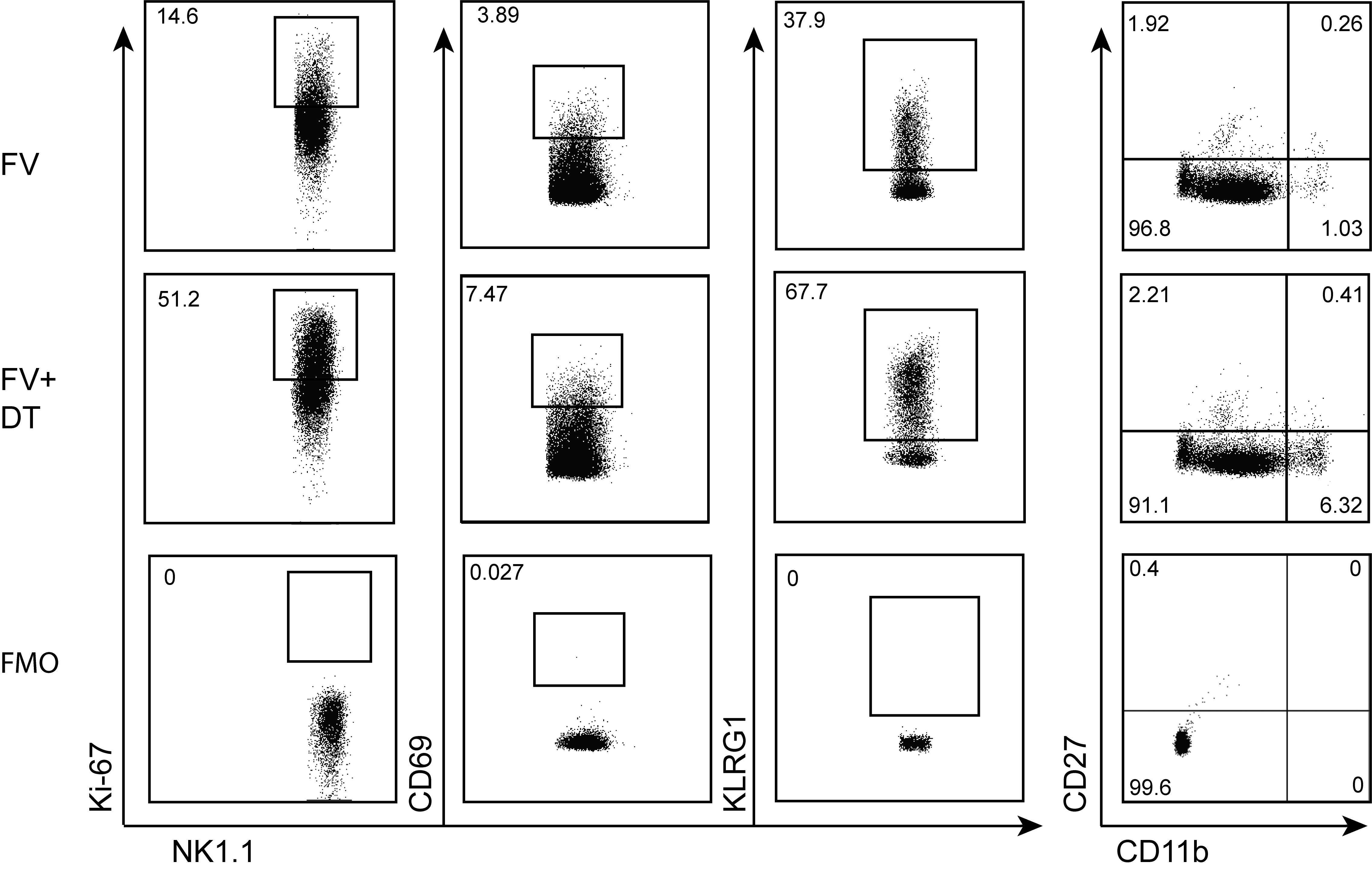

Supplement: Additional file 2: — Representative dotplots of activated, matured and proliferated NK cells Splenocytes were isolated from FV-infected mice (12 dpi) and NK cells were analyzed by flow cytometry (NK1.1+CD49b+CD3− cells). NK cells were evaluated for the different, indicated markers (Ki-67, CD69, KLRG1, CD27, CD11b). In the first row, representative dot plots of NK cell markers are shown during FV-infected. In the middle, dot plots of NK cells from FV-infected as well as Treg-ablated mice are depicted. Fluorescence-minus-one controls (FMOs) were performed for every staining (third row). [file 12977_2015_191_MOESM2_ESM.tif]

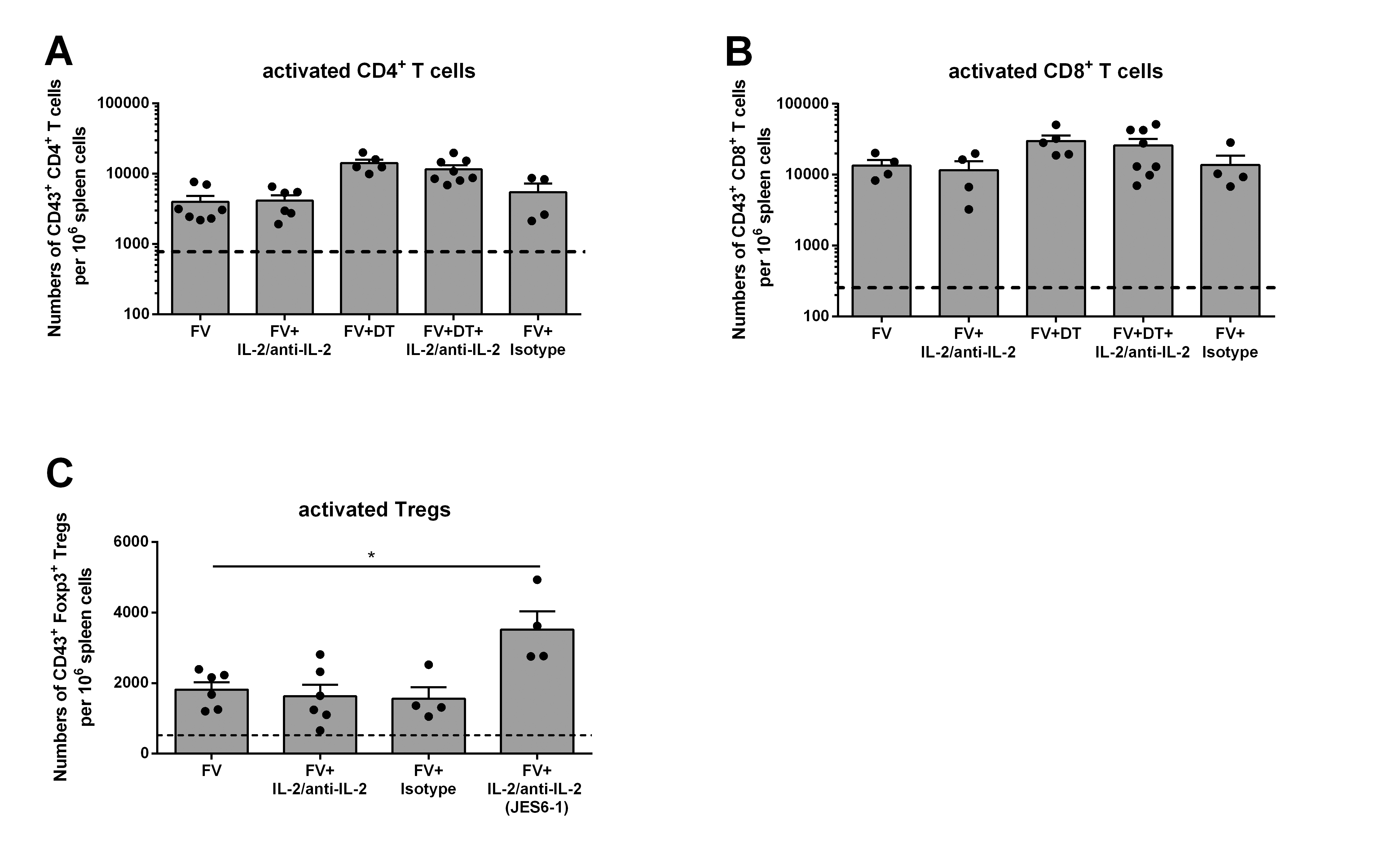

Supplement: Additional file 4: — Influence of IL-2/anti-IL-2 mAb complex treatment on T cell subsets Mice were infected with 20.000 SFFU of FV and sacrificed at 12 dpi. If indicated, Tregs were depleted by repeated injections of DT and mice were stimulated by injections of IL-2/anti-IL-2 mAb complex. Control mice were inoculated with isotype control. Single cell suspensions of splenocytes were stained for characteristic T cell markers ((a) CD4+ T cells; (b) CD8+ T cells and (c) Tregs) as well as activation (CD43) was analyzed using flow cytometry. Tregs were also stimulated with IL-2/anti-IL-2 mAb complex targeting CD25 on cell surface of Tregs (JES6-1) (c). Dotted lines represent the mean activation in naive mice. At least four mice per group were analyzed. Statistically significant differences between FV and FV+IL2/anti-IL-2 (JES6-1) were analyzed by Mann-Whitney test and indicated by * for p < 0.05. [file 12977_2015_191_MOESM4_ESM.tif]
